# Janus Kinase Inhibitors and Body Weight: Current Evidence and Potential

**DOI:** 10.3390/life16040667

**Published:** 2026-04-14

**Authors:** Krasimir Kraev, Yordanka Basheva-Kraeva, Maria Uchikova, Petar Uchikov, Bozhidar Hristov, Siyana Valova, Mladen Doykov, Desislav Stanchev, Atanas Boyukliev, Zguro Batalov

**Affiliations:** 1 Department of Propedeutics of Internal Diseases, Medical Faculty, Medical University of Plovdiv, 4000 Plovdiv, Bulgaria; 2 Department of Ophtalmology, Medical Faculty, Medical University of Plovdiv, 4000 Plovdiv, Bulgaria; 3 Department of Otorhynolaryngology, Medical Faculty, Medical University of Plovdiv, 4000 Plovdiv, Bulgaria; 4 Department of Special Surgery, Faculty of Medicine, Medical University of Plovdiv, 4000 Plovdiv, Bulgaria; 5 Section “Gastroenterology”, Second Department of Internal Diseases, Medical Faculty, Medical University of Plovdiv, 4000 Plovdiv, Bulgaria; 6 Section “Nephrology”, Second Department of Internal Diseases, Medical Faculty, Medical University of Plovdiv, 4000 Plovdiv, Bulgaria; 7 Department of Urology and General Medicine, Medical Faculty, Medical University of Plovdiv, 4000 Plovdiv, Bulgaria

**Keywords:** Janus kinase inhibitors, JAK–STAT pathway, weight gain, adipose tissue, metabolism

## Abstract

Janus kinase (JAK) inhibitors have become an important therapeutic option for a wide range of immune-mediated inflammatory diseases. By targeting intracellular cytokine signaling through inhibition of the JAK–STAT pathway, these agents provide effective suppression of multiple inflammatory cascades. Alongside their growing clinical use, changes in body weight—particularly weight gain—have recently been reported in clinical practice. Although this phenomenon has not consistently emerged as a prominent adverse event in randomized clinical trials, observational studies and real-world data suggest that weight gain may occur in some of the treated patients. The mechanisms underlying these changes remain barely understood and are likely multifactorial. Effective suppression of systemic inflammation may reverse inflammation-driven catabolism and restore metabolic balance, contributing to increases in body weight and lean body mass. In addition, experimental evidence indicates that JAK–STAT signaling participates in adipocyte differentiation, lipid metabolism, and energy regulation. Pharmacologic inhibition of this pathway may therefore influence adipose tissue biology, thermogenic activity, and appetite regulation through leptin-dependent signaling pathways. This review summarizes current evidence regarding weight and body composition changes associated with JAK inhibitor therapy, integrating findings from experimental studies, clinical trials, and real-world observations. Potential biological mechanisms are discussed alongside patient-related and disease-related factors that may modify the risk of weight gain. A better understanding of these immune–metabolic interactions may help guide clinical monitoring and future research on the metabolic consequences of JAK inhibition.

## 1. Introduction

Janus kinase (JAK) inhibitors have substantially broadened the therapeutic options for immune-mediated inflammatory diseases [1,2,3,4,5]. By interfering with intracellular cytokine signaling through inhibition of the JAK–STAT (Janus kinase–signal transducer and activator of transcription) pathway, these targeted synthetic agents possess effects across multiple inflammatory cascades simultaneously [6]. Over the last decade, their indications have steadily expanded to include rheumatoid arthritis, spondyloarthritis, atopic dermatitis, inflammatory bowel disease, and a range of hematologic and dermatologic conditions. Practical advantages such as oral administration and relatively rapid clinical response have further supported their increasing integration into routine practice [2,3,4,5].

JAK inhibitors are characterized by a distinct and still evolving safety profile [7,8,9]. Data from pharmacovigilance analyses and registry studies consistently highlight an increased risk of infections, cardiovascular and thrombotic events in selected populations, along with laboratory abnormalities [8,9,10]. Established concerns include infectious complications, laboratory abnormalities, and an increased risk of cardiovascular and thrombotic events in selected populations [7,10]. In parallel, changes in body weight and composition—particularly weight gain—have been reported with growing frequency in clinical practice. Although this phenomenon has not consistently emerged as a major adverse event in randomized controlled trials, real-world observations, cohort studies, and individual case reports suggest that it may be more prevalent and clinically relevant than initially appreciated.

Interpreting weight gain in patients with chronic inflammatory disease (CID) is very complex. Persistent systemic inflammation is known to disrupt metabolic homeostasis, contributing to increased resting energy expenditure, muscle catabolism, and altered fat distribution [11,12,13]. In rheumatoid arthritis, these processes may culminate in rheumatoid cachexia or sarcopenic obesity, where loss of lean mass occurs alongside increased adiposity [11]. When inflammation is effectively suppressed, partial reversal of these abnormalities may follow. Consequently, weight gain observed during JAK inhibitor therapy may, at least in part, reflect recovery from inflammation-driven catabolism rather than a direct adverse drug effect.

Nevertheless, accumulating experimental and translational data indicate that the JAK–STAT pathway itself plays a role in metabolic regulation [14,15,16,17,18]. This signaling network has been implicated in adipocyte differentiation, lipid handling, immune–adipose tissue crosstalk, and broader aspects of energy balance. Pharmacologic inhibition of these pathways could therefore influence fat storage, lipolysis, or thermogenic activity independently of anti-inflammatory effects. The extent to which such mechanisms operate in humans receiving JAK inhibitors, however, remains insufficiently defined.

Clinical evidence addressing this issue remains heterogeneous [19]. Reported rates of weight gain vary widely depending on disease indication, treatment duration, study design, and methods used to assess weight change. Most available data rely on crude measures such as body weight or body mass index, while detailed analyses of body composition, fat distribution, or metabolic consequences are rarely performed [20,21]. This limitation complicates interpretation and hinders meaningful counseling regarding the clinical significance of observed weight changes.

In this review, we summarize current evidence on weight and body composition changes associated with JAK inhibitor therapy, drawing on data from clinical trials, observational studies, and case reports. Potential biological mechanisms are examined alongside patient- and disease-related modifiers of risk. By placing these findings within the broader context of immune–metabolic interactions, we aim to provide a balanced framework for understanding and managing this increasingly recognized issue in inflammatory disease treatment.

## 2. Obesity, Adipose Tissue, and Immune–Metabolic Interactions

Obesity is most commonly defined by an excess of body fat and, in everyday clinical practice, is typically classified using body mass index (BMI). While convenient, this anthropometric approach captures only part of the underlying biological complexity. A growing body of evidence suggests that obesity is better understood as a disorder of adipose tissue function rather than simply a quantitative increase in fat mass [15,16]. Alterations in endocrine signaling, immune cell composition, and systemic metabolic regulation are central to this process and are particularly relevant when weight changes occur during immunomodulatory treatment [15,16,17].

Adipose tissue is increasingly recognized as an active endocrine and immunologically dynamic organ rather than a passive reservoir for energy storage. In addition to storing triglycerides, adipose tissue secretes a broad spectrum of adipokines, cytokines, chemokines, and lipid mediators that regulate insulin sensitivity, vascular function, and systemic inflammatory tone [15,16]. These mediators include classical adipokines such as leptin, adiponectin, and resistin, as well as pro-inflammatory cytokines including tumor necrosis factor-α (TNF-α), interleukin-6 (IL-6), and interleukin-1β (IL-1β). In addition, adipose tissue produces chemokines such as monocyte chemoattractant protein-1 (MCP-1/CCL2) and CCL5, which play a role in immune cell recruitment and local inflammatory amplification. Importantly, adipocytes coexist with diverse immune cell populations—including macrophages, T lymphocytes, B lymphocytes, and stromal cells—which together shape local and systemic metabolic responses [16,17]. Through these interactions, adipose tissue plays a central role in the interface between metabolic regulation and immune activation [17].

Not all adipose tissue behaves in the same way. White adipose tissue (WAT), which represents the predominant form in adults, is primarily located in subcutaneous depots and intra-abdominal (visceral) regions and serves as the main energy reservoir. Brown adipose tissue (BAT), found mainly in the supraclavicular, cervical, and paravertebral regions in adults, is specialized for heat production through uncoupled mitochondrial respiration. Between these two extremes are beige or “brite” adipocytes, which can emerge within white adipose depots—particularly in subcutaneous tissue—in response to physiological or environmental stimuli [19]. White adipose tissue primarily serves as an energy reservoir, whereas brown adipose tissue is specialized for heat production through uncoupled mitochondrial respiration. Between these two extremes are beige or “brite” adipocytes, which can emerge within white adipose depots in response to physiological or environmental stimuli [19]. The balance between these adipose tissue subtypes, as well as their functional state, influences energy expenditure and susceptibility to metabolic dysfunction or weight gain [19].

Chronic low-grade inflammation within adipose tissue represents a defining feature of obesity. This process is driven in part by increased immune cell infiltration, most notably macrophages that adopt a pro-inflammatory phenotype. Adipocyte hypertrophy, local hypoxia, and cellular stress further amplify cytokine production, creating a self-sustaining inflammatory environment [15,16]. Many mediators involved in this inflammatory response signal through intracellular kinase pathways that overlap with classical immune signaling networks, including the Janus kinase–signal transducer and activator of transcription (JAK–STAT) pathway [17].

At the same time, chronic inflammatory diseases such as rheumatoid arthritis frequently alter body composition in ways that are not immediately apparent from body weight alone. Ongoing systemic inflammation promotes muscle catabolism, increases resting energy expenditure, and disrupts lipid metabolism [11,12]. These metabolic alterations may result in sarcopenia or rheumatoid cachexia, conditions characterized by reduced lean mass and increased adiposity despite relatively stable body weight [11]. This inflammatory and catabolic state provides essential context when interpreting weight changes that occur following effective immunosuppressive therapy [12,13,14].

The interaction between inflammation and adipose tissue is therefore bidirectional. Adipose-derived cytokines and adipokines contribute to systemic inflammation, while inflammatory signaling pathways can alter adipocyte function and energy metabolism [15,16,17]. Interventions that suppress inflammatory signaling may therefore restore anabolic balance, modify adipose tissue biology, and alter energy expenditure [12,13,14]. Whether these changes manifest predominantly as fat accumulation, recovery of lean mass, or a combination of both is likely influenced by disease activity, baseline body composition, and treatment duration [22,23].

Within this broader framework, signaling pathways that link immune regulation and metabolic control deserve particular attention. The JAK–STAT pathway occupies a central position at this interface, given its role in cytokine signaling and its involvement in adipose tissue biology and metabolic regulation [17,18]. A clearer understanding of JAK–STAT–mediated effects on lipid metabolism and energy homeostasis is therefore essential for interpreting the metabolic changes observed during pharmacologic JAK inhibition [17,18,19].

## 3. JAK–STAT Signaling and Lipid Metabolism

The Janus kinase–signal transducer and activator of transcription (JAK–STAT) pathway represents a central intracellular signaling mechanism through which numerous cytokines, growth factors, and hormones exert their biological effects. In mammals, this signaling system involves four kinases—JAK1, JAK2, JAK3, and TYK2—that associate with type I and type II cytokine receptors at the cell membrane [6,17]. Ligand binding induces receptor dimerization and activation of receptor-associated JAKs, followed by phosphorylation of STAT transcription factors. Activated STAT dimers subsequently translocate to the nucleus, where they regulate gene expression involved in immune responses, cell survival, and metabolic regulation [6,17].

Although initially characterized as a key pathway in immune signaling, the JAK–STAT system is increasingly recognized as an important regulator of metabolic homeostasis. Components of this pathway are active in multiple metabolically relevant tissues, including adipose tissue, liver, and skeletal muscle [17,18]. Several STAT family members—particularly STAT1, STAT3, STAT5A, and STAT5B—are expressed in adipocytes and participate in processes such as adipocyte differentiation, lipid storage, insulin responsiveness, and energy balance [17,18].

Within adipose tissue, JAK–STAT signaling can be activated by a variety of cytokines and hormones, including interleukin-6, interferons, leptin, growth hormone, and prolactin [24,25,26,27]. Through these signaling inputs, the pathway influences both adipocyte-intrinsic functions and interactions between adipocytes and immune cells within the stromal vascular fraction [17,18]. Experimental studies have highlighted the role of STAT5 in adipogenesis and lipid storage, whereas STAT3 appears more closely associated with inflammatory signaling and insulin sensitivity in adipose tissue [17].

The influence of JAK–STAT signaling extends beyond white adipose tissue. Evidence suggests that this pathway may also regulate brown and beige adipocytes, which are responsible for thermogenic energy expenditure [19]. Experimental inhibition of JAK signaling has been shown to promote browning of white adipose tissue and improve metabolic parameters in animal models of obesity [19]. These findings provide a mechanistic framework through which alterations in JAK–STAT signaling could influence body weight regulation.

Mechanistically, the regulation of brown and beige adipose tissue involves complex interactions between cytokine signaling, mitochondrial function, and transcriptional control of thermogenic genes. Key regulators include uncoupling protein 1 (UCP1), peroxisome proliferator-activated receptor gamma coactivator 1-alpha (PGC-1α), and PR domain containing 16 (PRDM16), which drive thermogenic differentiation and mitochondrial biogenesis. JAK–STAT signaling has been shown to interact with these pathways, particularly through STAT3-mediated effects on mitochondrial activity and inflammatory signaling within adipose tissue [17,18]. Inhibition of JAK signaling in preclinical models has been associated with enhanced browning of white adipose tissue and increased energy expenditure, although these findings have not been consistently replicated in human studies. The net effect of JAK inhibition on thermogenesis in clinical settings therefore remains uncertain and may depend on disease context and systemic inflammatory status [19].

In addition to its role in adipose tissue biology, the JAK–STAT pathway contributes to the metabolic consequences of chronic inflammation. Pro-inflammatory cytokines that signal through JAK-dependent receptors promote a catabolic state characterized by increased resting energy expenditure and skeletal muscle breakdown [11,12,13]. Sustained activation of these pathways in CIDs contributes to muscle wasting and altered body composition [11,12,13]. From this perspective, pharmacologic inhibition of JAK–STAT signaling has the potential not only to suppress inflammation but also to modify inflammation-associated metabolic disturbances.

These considerations are particularly relevant in immune-mediated inflammatory diseases, where metabolic abnormalities frequently coexist with systemic inflammation. Restoration of inflammatory control through targeted therapies may therefore lead to shifts in energy balance and body composition [24,25,26,27]. Distinguishing between direct metabolic effects of JAK–STAT inhibition and indirect effects resulting from reduced inflammatory burden remains challenging and continues to be an area of active investigation [17,18,19].

Taken together, the available evidence supports an important role for the JAK–STAT pathway in the regulation of lipid metabolism, adipose tissue function, and systemic energy homeostasis. Its dual involvement in immune and metabolic signaling provides a biologically plausible explanation for changes in body weight observed during treatment with JAK inhibitors [17,18,19].

Beyond its role in adipocyte differentiation and inflammatory signaling, the JAK–STAT pathway also intersects with key enzymatic processes regulating lipid turnover. Lipolysis in adipocytes is primarily mediated by adipose triglyceride lipase (ATGL), hormone-sensitive lipase (HSL), and monoacylglycerol lipase (MGL), which sequentially hydrolyze triglycerides into free fatty acids and glycerol [16,17,18]. The activity of these enzymes is tightly regulated by hormonal signals, particularly catecholamines and insulin, as well as by lipid droplet–associated proteins such as perilipin. Experimental data suggest that cytokines signaling through JAK–STAT pathways may modulate lipolytic activity indirectly by influencing inflammatory tone and insulin sensitivity within adipose tissue [17].

Conversely, lipogenesis is driven by enzymes such as fatty acid synthase (FAS) and lipoprotein lipase (LPL), which facilitate fatty acid synthesis and uptake. Insulin signaling plays a central role in promoting lipogenesis, and disturbances in insulin receptor signaling—such as those observed in insulin resistance and hyperinsulinemia—can alter lipid storage and distribution [24,25,26,27]. Given that several cytokines acting through JAK-dependent pathways (e.g., IL-6, interferons) are implicated in insulin resistance, modulation of JAK–STAT signaling may influence the balance between lipolysis and lipogenesis.

Adiponectin is a key adipokine with anti-inflammatory, insulin-sensitizing, and cardioprotective properties, and plays a central role in metabolic homeostasis. In contrast to many other adipokines, adiponectin levels are typically reduced in obesity and chronic inflammatory states, where they are associated with insulin resistance, endothelial dysfunction, and increased cardiovascular risk [15,16].

The relationship between JAK–STAT signaling and adiponectin regulation is complex and not yet fully elucidated. Pro-inflammatory cytokines that signal through JAK-dependent pathways—particularly interleukin-6 (IL-6) and interferons—have been shown to suppress adiponectin expression in adipocytes, linking inflammatory signaling to adverse metabolic profiles [17,18]. Through these mechanisms, chronic activation of JAK–STAT signaling may contribute to reduced adiponectin levels observed in inflammatory diseases.

Pharmacologic inhibition of JAK signaling could theoretically reverse this effect by attenuating cytokine-driven suppression of adiponectin. Indirect evidence from studies of anti-inflammatory therapies suggests that improved inflammatory control may be associated with increases in adiponectin levels and improved insulin sensitivity [15,16]. However, direct clinical data evaluating adiponectin dynamics during JAK inhibitor therapy remain limited.

In addition to its systemic metabolic effects, adiponectin may influence adipose tissue function and energy balance through activation of AMP-activated protein kinase (AMPK) and peroxisome proliferator-activated receptor alpha (PPAR-α) pathways. These signaling cascades promote fatty acid oxidation, improve insulin sensitivity, and may counterbalance lipogenic and inflammatory processes within adipose tissue. The extent to which JAK inhibition modulates these adiponectin-mediated pathways represents an important area for future research.

Overall, adiponectin provides an additional link between immune signaling and metabolic regulation, further highlighting the complexity of immune–metabolic interactions during JAK inhibitor therapy.

## 4. Mechanism of Action of JAK Inhibitors and Potential Metabolic Consequences

JAK inhibitors are small-molecule agents designed to interfere with intracellular cytokine signaling by inhibiting the catalytic activity of one or more JAK family members. These compounds bind to the adenosine triphosphate (ATP)–binding site of JAKs, thereby preventing phosphorylation of downstream STAT proteins and attenuating cytokine-driven transcriptional responses [6]. In contrast to biologic agents that target individual cytokines or receptors, JAK inhibitors act at a shared intracellular signaling node, allowing broader modulation of inflammatory pathways [6].

Currently available JAK inhibitors differ in their selectivity for specific kinases. Tofacitinib primarily inhibits JAK1 and JAK3 with partial activity against JAK2, whereas baricitinib predominantly targets JAK1 and JAK2. More selective agents such as upadacitinib and abrocitinib preferentially inhibit JAK1, while ruxolitinib inhibits both JAK1 and JAK2 [1,6]. These pharmacologic differences influence downstream cytokine signaling and may contribute to variations in both therapeutic efficacy and adverse effect profiles [2,3,4,5].

One potential explanation for weight gain observed during JAK inhibitor therapy involves the reversal of inflammation-driven catabolism. Chronic inflammatory states promote muscle protein breakdown, increase resting energy expenditure, and interfere with anabolic metabolic signaling [11,12,13]. By suppressing cytokine signaling, JAK inhibitors may restore anabolic balance and reduce inflammation-induced metabolic stress, leading to recovery of lean body mass and normalization of energy expenditure [12,13,14].

In addition to these indirect effects, JAK inhibition may directly influence adipose tissue biology. The JAK–STAT pathway participates in adipocyte differentiation, lipid metabolism, and insulin signaling, suggesting that pharmacologic inhibition of this pathway could alter adipocyte function [17,18]. Experimental studies have shown that disruption of JAK signaling in adipocytes can influence lipolysis, lipid storage, and systemic metabolic regulation [18]. Such mechanisms may contribute to changes in body composition observed during JAK inhibitor therapy.

Effects on thermogenic regulation represent another potential mechanism. Cytokines that signal through JAK-dependent receptors have been implicated in the regulation of brown and beige adipose tissue activity and energy expenditure [19]. Inhibition of these pathways could theoretically reduce thermogenesis and favor positive energy balance, although the clinical significance of this mechanism remains uncertain [19].

Another pathway of interest involves leptin-mediated regulation of appetite and energy homeostasis. JAK2 plays a critical role in leptin receptor signaling within the hypothalamus [20,21,22]. Experimental studies demonstrate that leptin activates intracellular signaling cascades through JAK2-dependent mechanisms, influencing appetite regulation and energy balance [20,21]. Disruption of these signaling pathways may therefore alter satiety signaling and contribute to changes in body weight [22,23].

Genetic variability may also contribute to individual differences in metabolic responses to JAK inhibition. Variations in genes involved in JAK–STAT signaling or downstream metabolic pathways could influence susceptibility to weight gain during treatment [18]. However, the clinical relevance of such mechanisms remains insufficiently defined and requires further investigation.

**Figure 1:**
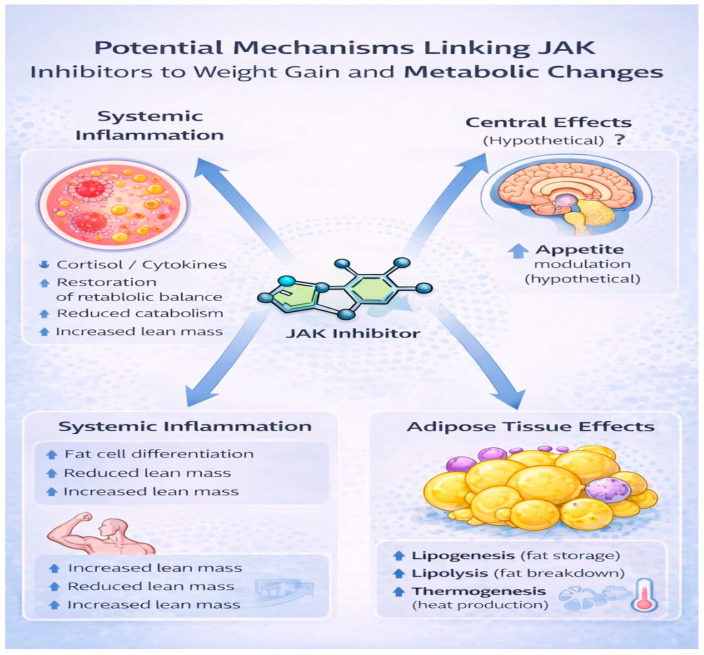
Potential mechanisms linking JAK inhibitor therapy and weight gain.

Importantly, the metabolic effects of JAK inhibition are unlikely to be uniform across patient populations. Baseline inflammatory burden, nutritional status, body composition, and concomitant therapies—particularly glucocorticoids—can all influence the metabolic response to treatment [24,25,26,27,28,29]. Consequently, weight gain during therapy should be interpreted within the broader clinical context rather than attributed solely to direct pharmacologic effects. The potential mechanisms linking JAK inhibition and body weight changes are summarized in Figure 1. A summary of the proposed mechanisms and supporting evidence is presented in Table 1.

Taken together, current evidence supports a model in which JAK inhibition influences body weight through both indirect effects—primarily via suppression of inflammation and reversal of catabolic metabolism—and potential direct effects on adipocyte signaling, lipid metabolism, and neuroendocrine regulation. However, the relative contribution of these mechanisms in humans remains incompletely defined.

## 5. Evidence from Clinical Trials and Observational Studies

Having discussed the biological mechanisms that may contribute to weight changes during JAK inhibitor therapy, it is important to examine the clinical evidence supporting these observations.

Randomized controlled trials evaluating JAK inhibitors have generally not identified weight gain as a frequent or prominent adverse event. Across pivotal trials in rheumatologic, dermatologic, and gastrointestinal indications, changes in body weight were often not prespecified outcomes and were therefore not systematically analyzed [7,24]. When reported, weight changes were typically recorded as unsolicited adverse events, a methodological approach that may underestimate their true incidence [7,8]. Long-term safety analyses of JAK inhibitors, including integrated datasets from large clinical trials, have similarly not highlighted weight gain as a dominant safety signal [7].

Where reported, weight gain in randomized trials appears to affect only a minority of treated patients and is usually modest in magnitude [24]. A systematic review and meta-analysis of oral JAK inhibitors reported weight gain in approximately 5–7% of treated individuals, although the magnitude of weight change varied considerably across studies [24]. Importantly, most clinical trials were designed with follow-up periods of one year or less and were not structured to detect gradual metabolic changes that might emerge during long-term therapy [7,24].

Differences between individual JAK inhibitors have been suggested but remain difficult to interpret. Trials involving ruxolitinib, particularly in hematologic indications, tend to report weight gain more frequently than studies involving other agents [30,31,32]. In contrast, studies of tofacitinib and JAK1-selective inhibitors have generally described lower rates of weight gain [7,25]. Whether these differences reflect drug-specific metabolic effects, disease-related factors, or variations in trial populations remains uncertain [24,25].

Overall, evidence from randomized trials suggests that clinically meaningful weight gain is neither universal nor an early hallmark of JAK inhibitor therapy [7,24]. However, the limited emphasis on metabolic outcomes within trial design warrants caution when extrapolating these findings to routine clinical practice [7,8].

### 5.1. Observational Studies and Real-World Data

In contrast to randomized trials, observational studies and real-world cohorts more consistently report weight gain during JAK inhibitor treatment. Several retrospective and prospective studies in patients with rheumatoid arthritis have documented increases in body weight or body mass index following initiation of tofacitinib therapy [25,28]. In one cohort study evaluating patients with rheumatoid arthritis receiving tofacitinib, measurable increases in BMI and visceral adiposity indices were observed over time [28].

Real-world studies have also reported changes in body composition and metabolic parameters during treatment with JAK inhibitors. Experimental and clinical investigations have demonstrated alterations in glucose metabolism and body composition in patients receiving tofacitinib, suggesting that metabolic effects may accompany suppression of inflammatory signaling [29]. These findings support the hypothesis that JAK inhibition may influence metabolic regulation beyond its anti-inflammatory effects [17,18,29].

In most observational cohorts, weight gain is modest for the majority of patients. However, a smaller subgroup appears to experience more substantial increases in body weight [24,25]. Several studies report that a subset of patients may gain more than 10% of their baseline body weight during treatment, although the proportion of affected individuals varies across cohorts [25]. Baseline inflammatory burden and body composition appear to influence the likelihood and magnitude of weight gain [11,25].

These observations support the hypothesis that weight gain may partly reflect reversal of inflammation-driven catabolism rather than a direct obesogenic effect of JAK inhibition [11,12,13]. Improvements in disease activity, mobility, and nutritional status following effective therapy may also contribute to changes in body weight observed in real-world settings [25,27].

The effect of JAK inhibitors on visceral adipose tissue (VAT) has also been explored, given its close association with cardiometabolic risk. Limited observational data suggest that treatment—particularly with tofacitinib—may be associated with increases in indices of visceral adiposity, including the visceral adiposity index and waist circumference in some patient cohorts [28]. These findings indicate that weight gain during therapy may involve not only overall increases in body mass but also changes in fat distribution toward metabolically active visceral depots.

However, these observations should be interpreted with caution. Changes in VAT may partly reflect recovery from inflammation-driven catabolism, as chronic inflammatory diseases are often associated with altered body composition and reduced fat mass. Restoration of metabolic balance following effective disease control may therefore contribute to expansion of adipose tissue stores. The clinical significance of these changes remains uncertain, and further studies incorporating imaging-based assessment of fat distribution are needed.

Evidence regarding changes in body composition during JAK inhibitor therapy remains limited. A small number of clinical studies have reported alterations in parameters such as visceral adiposity indices and metabolic markers, suggesting that weight gain may be accompanied by changes in fat distribution or metabolic function [28,29]. However, direct assessments using imaging-based techniques or detailed body composition analysis are scarce. Consequently, establishing a causal relationship between JAK inhibition and specific changes in fat mass or lean mass remains challenging, as improvements in disease activity, physical function, and nutritional intake may also contribute.

### 5.2. Case Series and Pharmacovigilance Reports

Case reports and pharmacovigilance analyses provide additional insight into atypical or more pronounced weight changes associated with JAK inhibitor therapy [8,9]. Conference reports and registry data also suggest that clinically relevant weight gain may occur in selected patients treated with JAK inhibitors for rheumatoid arthritis [33]. Several case series have described substantial weight gain in individual patients treated with tofacitinib, with reported increases ranging from 5 to 19 kg [34]. Although uncommon, these cases illustrate that clinically meaningful weight gain may occur in selected individuals.

Spontaneous pharmacovigilance reports and post-marketing surveillance databases have also identified weight gain as a potential adverse event associated with JAK inhibitor therapy [8,9]. While such data are subject to reporting bias and lack standardized measurements, they may capture metabolic effects not detected in controlled clinical trials [8].

Studies in hematologic diseases provide further evidence that JAK inhibition may influence body weight. In patients with myelofibrosis treated with ruxolitinib or fedratinib, increases in body weight and improvements in metabolic parameters have been reported [34,35,36]. These changes have been interpreted as reflecting both disease-related metabolic recovery and potential drug-specific metabolic effects [37,38].

### 5.3. Summary of Clinical Evidence

Taken together, available clinical evidence indicates that weight gain during JAK inhibitor therapy is heterogeneous in both frequency and magnitude [39]. Randomized trials suggest a relatively low incidence of weight gain, whereas observational studies and real-world cohorts report weight increases more frequently [40,41,42]. Importantly, weight changes appear to be influenced by baseline inflammatory status, body composition, and treatment indication [30,35].

These findings highlight the importance of interpreting weight changes within the broader clinical context rather than viewing them solely as adverse drug reactions [24,27,43]. In some patients, moderate weight gain may reflect restoration of metabolic balance following suppression of chronic inflammation [44,45].

Across available studies, the reported prevalence of weight gain during JAK inhibitor therapy varies considerably depending on study design and population, but is generally estimated at approximately 5–10% of treated patients [24,25]. In most cases, weight increases are modest, typically within a range of 1–3 kg during the first year of treatment. However, a smaller subgroup of patients may experience more pronounced weight gain, occasionally exceeding 10% of baseline body weight [25]. These findings suggest that while weight gain is not a universal effect, it represents a clinically relevant phenomenon in a subset of patients.

## 6. Clinical Interpretation and Integrative Perspectives

The relationship between JAK inhibitor therapy and changes in body weight represents a complex intersection of immune regulation and metabolic physiology. Evidence from clinical trials, observational cohorts, and mechanistic studies suggests that weight gain during treatment is neither universal nor uniform across patient populations. Rather, it appears to result from the interplay of several biological and clinical factors, including suppression of systemic inflammation, alterations in adipose tissue biology, and potential neuroendocrine effects.

One of the most plausible explanations for weight gain observed during JAK inhibitor therapy is the reversal of inflammation-driven catabolism. Chronic inflammatory diseases such as rheumatoid arthritis are characterized by elevated resting energy expenditure, skeletal muscle breakdown, and alterations in lipid metabolism, which may lead to conditions such as rheumatoid cachexia or sarcopenic obesity. Effective suppression of pro-inflammatory cytokine signaling through JAK inhibition may restore anabolic balance, reduce energy expenditure, and promote recovery of lean body mass. In this context, moderate weight gain may represent an expected metabolic consequence of disease control rather than an adverse pharmacologic effect.

At the same time, experimental and translational evidence indicates that the JAK–STAT signaling pathway participates directly in metabolic regulation. Components of this pathway influence adipocyte differentiation, lipid handling, and immune–adipose tissue communication. Pharmacologic inhibition of these signaling networks could therefore modify adipocyte function and energy balance independently of anti-inflammatory effects. However, the extent to which these mechanisms contribute to weight gain in humans receiving JAK inhibitors remains incompletely understood.

Another potential contributor involves central regulation of appetite and energy homeostasis. The JAK2 pathway is involved in leptin receptor signaling within the hypothalamus, suggesting that interference with this pathway could theoretically influence satiety signaling. While this mechanism is biologically plausible, direct clinical evidence demonstrating altered appetite regulation during JAK inhibitor therapy remains limited. Consequently, this hypothesis should currently be regarded as speculative and warrants further investigation.

Importantly, clinical observations suggest that weight changes during JAK inhibitor therapy are heterogeneous. Most patients experience modest increases in body weight, typically within the range of several kilograms during the first year of treatment. However, a subset of individuals appears to develop more pronounced weight gain. Differences in baseline inflammatory burden, nutritional status, body composition, and concomitant therapies—particularly glucocorticoids—likely contribute to this variability. These factors highlight the importance of considering individual patient characteristics when interpreting weight changes during treatment.

From a clinical perspective, the significance of weight gain associated with JAK inhibitors remains uncertain. In patients with severe inflammatory disease and low baseline body mass, weight gain may reflect restoration of metabolic balance and improvement in overall health status. Conversely, in individuals with pre-existing obesity or cardiometabolic risk factors, further weight gain could potentially contribute to adverse metabolic outcomes. Careful monitoring of body weight, metabolic parameters, and cardiovascular risk factors may therefore be appropriate in selected patients receiving long-term therapy.

An important clinical question is whether weight gain observed during JAK inhibitor therapy represents the development of obesity or a normalization of body weight following chronic inflammation. Available evidence suggests that, in most patients, weight gain remains within the range of modest increases and does not lead to overt obesity. In individuals with active inflammatory disease and features of cachexia or reduced lean mass, weight gain may reflect restoration of metabolic balance and improvement in nutritional status. However, in patients with pre-existing overweight or obesity, additional weight gain—particularly if accompanied by increased visceral adiposity—may have less favorable metabolic implications.

The interpretation of substantial weight gain in individual patients remains complex. While moderate increases in body weight may reflect beneficial recovery from inflammation-driven catabolism, more pronounced weight gain raises the possibility of disproportionate fat accumulation. This distinction is particularly important given the potential impact on cardiometabolic risk. Current evidence does not allow clear differentiation between adaptive metabolic recovery and adverse fat accumulation in such cases. Therefore, individualized clinical assessment—including evaluation of body composition, metabolic parameters, and cardiovascular risk factors—is essential when significant weight gain occurs during therapy.

Several limitations of the available evidence should also be acknowledged. Most randomized clinical trials were not designed to systematically evaluate metabolic outcomes, and weight changes were often reported as unsolicited adverse events. Observational studies provide valuable real-world insight but are frequently limited by small sample sizes, short follow-up durations, and potential confounding factors. Furthermore, most studies rely on crude anthropometric measures such as body weight or BMI, whereas detailed assessments of body composition, visceral adiposity, and metabolic function remain scarce.

Future research should aim to clarify the metabolic consequences of JAK inhibitor therapy through prospective studies incorporating comprehensive metabolic phenotyping. Such investigations should include detailed assessments of body composition, insulin sensitivity, lipid metabolism, and energy expenditure. Additionally, exploration of genetic and molecular factors that influence individual susceptibility to weight changes may help identify patient subgroups at higher risk. Improved understanding of these mechanisms could ultimately inform personalized therapeutic strategies and optimize long-term metabolic outcomes in patients receiving JAK inhibitors.

## 7. Conclusions

JAK inhibitors have transformed the management of multiple immune-mediated inflammatory diseases by providing effective, targeted suppression of cytokine signaling through the JAK–STAT pathway. As their clinical use continues to expand, a broader understanding of their systemic metabolic effects has become increasingly important. Emerging evidence suggests that changes in body weight, including weight gain in a subset of patients, may occur during treatment with these agents.

Current data indicate that weight gain associated with JAK inhibitor therapy is heterogeneous in both frequency and magnitude. In many patients, modest increases in body weight may reflect the reversal of inflammation-driven catabolism and restoration of metabolic balance following effective control of CID. At the same time, experimental and translational findings suggest that JAK–STAT signaling participates in adipose tissue biology, lipid metabolism, and energy regulation, raising the possibility that pharmacologic inhibition of this pathway could also exert direct metabolic effects.

Despite growing interest in this phenomenon, the available clinical evidence remains limited. Most studies rely on simple anthropometric measures, and detailed analyses of body composition, energy expenditure, and metabolic function are rarely performed. Consequently, the precise mechanisms underlying weight changes during JAK inhibitor therapy and their long-term clinical implications remain incompletely understood.

Future research should focus on prospective studies incorporating comprehensive metabolic assessments, including body composition analysis and metabolic profiling. Improved understanding of immune–metabolic interactions in the context of JAK inhibition will help clarify whether observed weight changes represent beneficial recovery from inflammatory catabolism, unintended metabolic consequences of therapy, or a combination of both. Such insights will ultimately support more informed clinical monitoring and individualized treatment strategies for patients receiving JAK inhibitors.

## Figures and Tables

**Table 1:** Biological mechanisms potentially contributing to weight changes during JAK inhibitor therapy.

Mechanism	Biological Pathway	Supporting Evidence	Key References
Reversal of inflammation-driven catabolism	Suppression of pro-inflammatory cytokine signaling reduces resting energy expenditure and muscle protein breakdown	Clinical observations in inflammatory diseases; improvement of rheumatoid cachexia following effective therapy	[11,12,13]
Direct effects on adipocyte metabolism	JAK–STAT signaling regulates adipocyte differentiation, lipid metabolism, and adipokine production	Experimental and molecular studies in adipocytes	[17,18]
Thermogenesis modulation	JAK signaling influences activity of brown and beige adipose tissue and energy expenditure	Preclinical models demonstrating adipose tissue browning and metabolic changes	[19]
Appetite regulation	JAK2-mediated leptin receptor signaling participates in hypothalamic appetite control	Experimental studies on leptin signaling pathways	[20,21,22]
Metabolic recovery following disease control	Reduced systemic inflammation improves nutritional status, physical activity, and metabolic balance	Observational clinical studies and real-world cohorts	[24,25,26,27]

## Data Availability

Data are available on request.

## References

[1] Heymann W.R. (2025). Janus kinase inhibitors battle in the heavyweight class. J. Am. Acad. Dermatol..

[2] Nash P., Kerschbaumer A., Dörner T., Dougados M., Fleischmann R.M., Geissler K., McInnes I., E Pope J., van der Heijde D., Stoffer-Marx M. (2021). Points to consider for the treatment of immune-mediated inflammatory diseases with Janus kinase inhibitors. Ann. Rheum. Dis..

[3] Cunningham K.N., Rosmarin D. (2023). Vitiligo treatments: Review of current therapeutic modalities and JAK inhibitors. Am. J. Clin. Dermatol..

[4] Gupta A.K., Wang T., Polla Ravi S., Bamimore M.A., Piguet V., Tosti A. (2023). Systematic review of newer agents for alopecia areata in adults: Janus kinase inhibitors, biologics and phosphodiesterase-4 inhibitors. J. Eur. Acad. Dermatol. Venereol..

[5] Dogra S., Sharma A., Mehta H., Sarkar R. (2023). Emerging role of topical Janus kinase inhibitors in dermatological disorders. Clin. Exp. Dermatol..

[6] Hodge J.A., Kawabata T.T., Krishnaswami S., Clark J.D., Telliez J.B., Dowty M.E., Zwillich S. (2016). The mechanism of action of tofacitinib-an oral Janus kinase inhibitor for the treatment of rheumatoid arthritis. Clin. Exp. Rheumatol..

[7] Cohen S.B., Tanaka Y., Mariette X., Curtis J., Lee E.B., Nash P., Winthrop K., Charles-Schoeman C., Wang L., Chen C. (2020). Long-term safety of tofacitinib up to 9.5 years: A comprehensive integrated analysis of the rheumatoid arthritis clinical development programme. RMD Open.

[8] Hoisnard L., Lebrun-Vignes B., Maury S., Mahevas M., El Karoui K., Roy L., Sbidian E. (2022). Adverse events associated with JAK inhibitors in 126,815 reports from the WHO pharmacovigilance database. Sci. Rep..

[9] Song Y.K., Song J., Kim K., Kwon J.W. (2022). Potential adverse events reported with the Janus kinase inhibitors approved for the treatment of rheumatoid arthritis using spontaneous reports and online patient reviews. Front. Pharmacol..

[10] Aymon R., Mongin D., Bergstra S.A., Choquette D., Codreanu C., De Cock D., Dreyer L., Elkayam O., Huschek D., Hyrich K.L. (2024). Evaluation of discontinuation for adverse events of JAK inhibitors and bDMARDs in an international collaboration of rheumatoid arthritis registers (the ‘JAK-pot’ study). Ann. Rheum. Dis..

[11] Hanaoka B.Y., Zhao J., Heitman K., Khan F., Jarjour W., Volek J., Gower B.A. (2022). Interaction effect of systemic inflammation and modifiable rheumatoid cachexia risk factors on resting energy expenditure in patients with rheumatoid arthritis. JCSM Clin. Rep..

[12] Bonetto A., Aydogdu T., Jin X., Zhang Z., Zhan R., Puzis L., Koniaris L.G., Zimmers T.A. (2012). JAK/STAT3 pathway inhibition blocks skeletal muscle wasting. Am. J. Physiol. Endocrinol. Metab..

[13] Chen Z., Li B., Zhan R.Z., Rao L., Bursac N. (2021). Exercise mimetics and JAK inhibition attenuate IFN-γ–induced wasting in engineered human skeletal muscle. Sci. Adv..

[14] Price F.D., von Maltzahn J., Bentzinger C.F., Dumont N.A., Yin H., Chang N.C., Wilson D.H., Frenette J., Rudnicki M.A. (2014). Inhibition of JAK-STAT signaling stimulates adult satellite cell function. Nat. Med..

[15] Ouchi N., Parker J.L., Lugus J.J., Walsh K. (2011). Adipokines in inflammation and metabolic disease. Nat. Rev. Immunol..

[16] Kirichenko T.V., Markina Y.V., Bogatyreva A.I., Tolstik T.V., Varaeva Y.R., Starodubova A.V. (2022). Adipokines in inflammatory mechanisms of obesity. Int. J. Mol. Sci..

[17] Richard A.J., Stephens J.M. (2011). Emerging roles of JAK-STAT signaling pathways in adipocytes. Trends Endocrinol. Metab..

[18] Shi S.Y., Luk C.T., Brunt J.J., Sivasubramaniyam T., Lu S.Y., Schroer S.A., Woo M. (2014). Adipocyte-specific deficiency of Janus kinase (JAK) 2 in mice impairs lipolysis and increases body weight, and leads to insulin resistance with ageing. Diabetologia.

[19] Qurania K.R., Ikeda K., Wardhana D.A., Barinda A.J., Nugroho D.B., Kuribayashi Y., Emoto N. (2018). Systemic inhibition of Janus kinase induces browning of white adipose tissue and ameliorates obesity-related metabolic disorders. Biochem. Biophys. Res. Commun..

[20] Kellerer M., Koch M., Metzinger E., Mushack J., Capp E., Häring H.U. (1997). Leptin activates PI-3 kinase in C2C12 myotubes via janus kinase-2 (JAK-2) and insulin receptor substrate-2 (IRS-2) dependent pathways. Diabetologia.

[21] Kellerer M., Lammers R., Fritsche A., Strack V., Machicao F., Borboni P., Häring H.U. (2001). Insulin inhibits leptin receptor signalling in HEK293 cells at the level of janus kinase-2: A potential mechanism for hyperinsulinaemia-associated leptin resistance. Diabetologia.

[22] Jiang L., Li Z., Rui L. (2008). Leptin stimulates both JAK2-dependent and JAK2-independent signaling pathways. J. Biol. Chem..

[23] Ibba L., Gargiulo L., Bianco M., Di Giulio S., Cascio Ingurgio R., Alfano A., Costanzo A. (2025). Potential role of leptin in Janus kinase 2 inhibitor-associated weight gain: A monocentric retrospective study. Clin. Exp. Dermatol..

[24] Xiong G., Yu E., Heung M., Yang J., Lowe M., Abu-Hilal M. (2024). Weight gain secondary to the use of oral Janus kinase inhibitors: A systematic review and meta-analysis. JAAD Int..

[25] Wollenhaupt J., Morel J., Daien C., Ruyssen-Witrand A., Lukas C., Richez C., Citera G. (2025). Analysis of the impact of tofacitinib treatment on weight and body mass index in patients with rheumatoid arthritis. ACR Open Rheumatol..

[26] Palmowski A., Buttgereit F., Simon D., Kleyer A., Drzeniek N.M. (2026). Tofacitinib-Induced Weight Gain in Context: Comment on the article by Wollenhaupt et al. ACR Open Rheumatol..

[27] Mehta P., Kharouf F., Abarza V.C., Gao S., Cook R.J., Poddubnyy D., Chandran V. (2025). Exploring the impact of conventional and targeted DMARDs on body weight in patients with PsA. Rheumatology.

[28] Novikova D.S., Udachkina H.V., Markelova E.I., Kirillova I.G., Misiyuk A.S., Demidova N.V., Popkova T.V. (2019). Dynamics of BMI and visceral adiposity index in RA patients treated with tofacitinib. Rheumatol. Int..

[29] Chikugo M., Sebe M., Tsutsumi R., Iuchi M., Kishi J., Kuroda M., Sakaue H. (2018). Effect of Janus kinase inhibition by tofacitinib on body composition and glucose metabolism. J. Med. Investig..

[30] Tremblay D., Cavalli L., Sy O., Rose S., Mascarenhas J. (2022). The effect of fedratinib, a selective inhibitor of janus kinase 2, on weight and metabolic parameters in patients with intermediate-or high-risk myelofibrosis. Clin. Lymphoma Myeloma Leuk..

[31] Sapre M., Tremblay D., Wilck E., James A., Leiter A., Coltoff A., Koshy A.G., Kremyanskaya M., Hoffman R., Mascarenhas J.O. (2019). Metabolic effects of JAK1/2 inhibition in myeloproliferative neoplasms. Sci. Rep..

[32] Breccia M., Bartoletti D., Bonifacio M., Palumbo G.A., Polverelli N., Abruzzese E., Palandri F. (2019). Impact of comorbidities and body mass index in patients with myelofibrosis treated with ruxolitinib. Ann. Hematol..

[33] Szeja N., Grosicki S. (2020). Refeeding syndrome in hematological cancer patients. Expert. Rev. Hematol..

[34] (2024). AB0633 Do Janus kinase inhibitors lead to weight gain in rheumatoid arthritis patients?. Ann. Rheum. Dis..

[35] Shah K., Shukla D., Patel M., Malhotra S. (2023). Case series on tofacitinib-induced weight gain. Indian J. Pharmacol..

[36] Ch’en P.Y., Ng J., Song E.J. (2023). Weight gain secondary to JAK inhibitors. Arch. Dermatol. Res..

[37] Panaccione R., Isaacs J.D., Chen L.A., Wang W., Marren A., Kwok K., Su C. (2021). Characterization of creatine kinase levels in tofacitinib-treated patients with ulcerative colitis: Results from clinical trials. Dig. Dis. Sci..

[38] Isaacs J.D., Zuckerman A., Krishnaswami S., Nduaka C., Lan S., Hutmacher M.M., Riese R. (2014). Changes in serum creatinine in patients with active rheumatoid arthritis treated with tofacitinib: Results from clinical trials. Arthritis Res. Ther..

[39] Serelis J., Kontogianni M.D., Katsiougiannis S., Bletsa M., Tektonidou M.G., Skopouli F.N. (2008). Effect of anti-TNF treatment on body composition and serum adiponectin levels of women with rheumatoid arthritis. Clin. Rheumatol..

[40] de Souza S., Williams R., Nikiphorou E. (2024). Clinician and patient views on janus kinase inhibitors in the treatment of inflammatory arthritis: A mixed methods study. BMC Rheumatol..

[41] Human A., Pagnoux C. (2019). Diagnosis and management of ADA2 deficient polyarteritis nodosa. Int. J. Rheum. Dis..

[42] Roskoski R. (2023). Deucravacitinib is an allosteric TYK2 protein kinase inhibitor FDA-approved for the treatment of psoriasis. Pharmacol. Res..

[43] Jacobsohn D.A., Margolis J., Doherty J., Anders V., Vogelsang G.B. (2002). Weight loss and malnutrition in patients with chronic graft-versus-host disease. Bone Marrow Transpl..

[44] Stafford M., Hemingway H., Marmot M. (1998). Weight fluctuation and physical functioning: Whitehall II Study. Int. J. Obes. Relat. Metab. Disord..

[45] Ji Y., Huang Q., Liu H., Phillips C. (2021). The Effect of Perceived Weight Change on Performance Evaluation and the Moderating Role of Anti-fat Bias. Front. Psychol..

